# Can interprofessional education change students’ attitudes? A case study from Lebanon

**DOI:** 10.1186/s12909-022-03608-z

**Published:** 2022-07-23

**Authors:** Carine J. Sakr, Lina Fakih, Jocelyn Dejong, Nuhad Yazbick-Dumit, Hussein Soueidan, Wiam Haidar, Elias Boufarhat, Imad Bou Akl

**Affiliations:** 1 Employee Health Unit, Department of Family Medicine, Faculty of Medicine, American University of Beirut Medical Center, 1107 2020 Beirut, Lebanon; 2 Faculty of Health Sciences, American University of Beirut, Riad El Solh, PO Box 11-0236, 1107 2020 Beirut, Lebanon; 3 Hariri School of Nursing, American University of Beirut, 1107 2020 Beirut, Lebanon; 4 American University of Beirut, 1107 2020 Beirut, Lebanon; 5 Department of Internal Medicine, Division of Pulmonary and Critical Care Medicine, American University of Beirut Medical Center, Beirut, Lebanon; 6 Department of Internal Medicine, AUBMC, 1107 2020 Beirut, Lebanon

**Keywords:** Attitudes, Collaborative learning, Interprofessional education, Interprofessional collaboration

## Abstract

**Background:**

Interprofessional collaboration is key to improving the health of individuals and communities. It is supported by provision of Interprofessional education (IPE) which has recently emerged in the Middle East region. This study investigated changes in healthcare students’ attitudes towards interprofessional collaboration after undertaking the Interprofessional Education and Collaboration (IPEC) course.

**Methods:**

A paper-based anonymous survey using the Interprofessional Attitude Scale (IPAS) was administered to a sample of 346 health students (nursing, medicine, and public health) pre/post undertaking the IPEC course. Less than half of the students provided a post response, with pre/post survey results of 111 pairs subsequently matched and analyzed.

**Results:**

Results showed elevated pre-course scores, an improvement in students’ attitudes towards the interprofessional biases domain of the IPAS, and a slight decline in their scores in the remaining 4 domains (team roles and responsibilities, patient centeredness, community centeredness, and diversity and ethics). These changes were not statistically significant, except for the patient centeredness domain (*p* = 0.003**).

**Conclusions:**

The study provided important results about attitudes towards interprofessional collaboration. These findings are essential because our institution is one of few in Lebanon that provides this mandatory course to a large group of health professionals. Future studies should investigate these changes in attitude scores in a larger sample size, and how these attitudes would influence collaboration post-graduation.

## Introduction

It is increasingly recognized that interprofessional collaboration and effective team building are needed to improve the health of individuals and populations. This interprofessional collaboration must begin in educational institutions. According to World Health Organization, interprofessional education (IPE) occurs when “students from two or more professions learn about, from, and with each other to enable effective collaboration and improve health outcomes” [1]. The Lancet Commission on Health professionals for a New Century identified the need for IPE to break down professional silos and to enhance collaborative and non-hierarchical relationships in health teams [2]. These changes have stimulated the introduction of IPE courses in North America and Europe [3]. This trend however is only beginning in the Middle East region and needs further research and encouragement. The first regional conference on IPE was held in Qatar in 2015. In Saudi Arabia, medical and dental students both agreed that interprofessional learning helped them develop respect, trust, and appreciation for other professions; however, students did not favor IPE for learning discrete professional identities and roles [4]. Students from many disciplines in Qatar (pharmacy, medicine, and public health) reported that IPE enhanced their communication skills, collaboration, and appreciation of professional roles [5]. In Lebanon, the Lebanese American University offered a five-step workshop-based series to students of multiple professions (nutrition, pharmacy, nursing, and medicine). After IPE participation, students reported being satisfied with the experience and showed enhanced readiness for interprofessional learning [6].

The core of IPE lies in training synergy among health workers and equipping them with collaborative skills needed for today’s complex environment [7]. However, the success of such an initiative is highly dependent on attitudes and readiness of healthcare students to this type of learning [8]. In fact, students’ attitudes have been identified as the most important factor in determining the success of IPE [9]. The attitude of individuals from one profession can highly impact their attitude and behavior towards individuals from another profession [10].

The aim of this study was to evaluate the attitudes of students at the American University of Beirut (AUB) towards interprofessional collaboration and to assess whether the recently introduced IPEC course impacted that attitude.

## Methods

### Implementation of IPE at the American University of Beirut (AUB)

At AUB, an IPE task force from the Faculty of Medicine, the School of Nursing, and the Faculty of Public Health was created in 2013 to introduce IPE and collaboration (IPEC) course into the three curricula. The impetus for the introduction of the IPEC was the hosting of the Lancet commission on health professionals’ education launch, on which a former Dean of the Faculty of Health Science had been represented.

Specifically, the IPEC committee introduced a new, required IPE course in 2016–2017 that combined students from the 3 mentioned specialties (Medicine, Nursing, and Public Health), and introduced them to the IPEC through case-based group discussions utilizing collaborative learning. The course is based on small groups working together on hypothetical cases requiring the input and role of all three specialties. The cases were designed by interprofessional teams of faculty members and focus both on individual and population health perspectives on specific health topics. Collaborative learning was adapted as an approach given its dual focus both on content and on building social skills (including such skills as communication, mutual respect, and team building). While students in each area are expected to have learned about the topic in their curriculum from the perspective of their own fields, the course necessitates collaboration among the fields. Essential skills such as understanding each other’s roles and responsibilities, teamwork, communication, and ethics are the focus of this course, as they are the cornerstones of competency-based IPE [11]. The competencies were inspired by the IPEC collaborative which now combines 21 different professional associations in the US [12].

The course typically involved 170 students, 90 from the Medical School, 40 from the Nursing School, and 40 from the faculty of Public Health. They were divided into groups of nine (4–5 Medicine, 2–3 Nursing, 2 MPH). Due to logistical reasons, the course was given twice a year, in the fall and spring semesters.

The course was a novel experience in many ways at the three concerned faculties. It was the first interprofessional course. It was also the first collaborative educational offering based on a small case-based discussion necessitating the input of the whole group.

Because of this novelty, multiple workshops were performed to train the faculty involved in developing the course and on the cases and running a collaborative learning experience. Faculty members from the three faculties who signed up for the course were also trained as group facilitators.

The implementation of the course faced many challenges ranging from curricular change of the three faculties involved, to securing adequate resources to train and organize the implementation of such a resource intensive course. The course involved 40 faculty members per year to facilitate the case-based discussions. The diversity of student knowledge background, experience in teamwork, expectations from the course, and their perspective of healthcare added to the level of difficulty in implementing the course. Five main topics were used to explicate Interprofessional Education and Collaboration during the course, and they included: smoking cessation, HIV testing, cardiac care, substance abuse, and care of the elderly. These were chosen as health topics that require interprofessional collaboration, are prominent across different stages of the life cycle, and for which there was faculty expertise.

## Survey design

We chose to utilize a quantitative survey-based study to assess the students’ attitudes toward the core competencies of interprofessional practice before and after the implementation of the newly developed IPEC course at AUB the first time it was taught.

The study included students in the three AUB faculties involved in the implementation of IPEC course in the academic years of 2016–2017 and 2017–2018. During these two academic years, 180 s year medical students, 80 fourth year nursing students and 86 Master of Public Health Students took the course. Students taking the IPEC course were asked to complete the survey at the beginning of the course and three months later at the completion of the course. The study was approved by the institutional review board (IRB) at the American University of Beirut.

A self-administered paper-based anonymous survey was created in English, the language of instruction. The first part of the survey contained a user-generated anonymous code to track each student’s responses pre and post IPEC course and allow us to do paired analysis of the pre and post survey. The second part included a validated survey instrument, the Interprofessional Attitude Scale (IPAS) with the permission of the authors. The IPAS is a 27-item instrument that assesses the five subdomains of teamwork: roles and responsibilities (nine questions), patient centeredness (five questions), interprofessional biases (three questions), diversity and ethics (four questions), and community centeredness (six questions). These five domains were inspired from the four competency domains in the 2011 IPEC competencies report [13]. these are: values/ethics for interprofessional practice, roles/responsibilities, interprofessional communication, and teams and teamwork. Values/ethics for interprofessional practice are important in shaping a professional identity, one that is both professional and interprofessional in nature [11]. The roles and responsibilities domain is an explicit feature of most interprofessional collaboration frameworks. It is essential for collaborative practice as it allows the understanding of how professional roles and responsibilities complement each other in a patient and community centered approach [11]. The interprofessional communication competency helps professionals in preparing for collaborative practice. The instrument also includes both a patient centeredness domain and a community centeredness domain since direct healthcare professionals and public health professionals are linked together, and share roles and responsibilities pertaining to the health of people [11].

The Likert scale was scored as follows: 1, strongly disagree; 2, disagree; 3, somewhat disagree; 4, neither agree nor disagree; 5, somewhat agree; 6, agree; and 7, strongly agree. According to the survey instructions, one question was reverse scored. The total score and sub-domain averages were calculated.

## Data collection

At the beginning of the first session and at the end of the last session of the IPEC course, paper-based surveys were provided to the student participants by the respective group facilitator. The first page included an informed consent explaining the purpose of the study, that participation is voluntary, and as such informed consent of participation was confirmed by completing the questionnaire. Although the first part of the survey contained a user-generated anonymous code to track pre and post responses of students for paired statistical analysis, the response and the code were in no way traceable back to the individual. The study results are reported in aggregate form.

## Data analysis

SPSS version 24 was used for the statistical analysis. The answers to the IPAS instrument were not analyzed individually but at the domain level, for the five domains covered. Categorical variables were presented using frequencies and percentages. Continuous variables were presented using means. The differences between students pre and post test scores was calculated using paired analysis. A *p*-value of 0.05 was considered statistically significant.

## Results

Table 1 describes the distribution of completed questionnaires. A total of 260 questionnaires were filled pre-IPE course, by students in the Faculty of Medicine, School of Nursing and Faculty of Health Sciences respectively, which represents a 75.1 overall response rate. Students completed 163 questionnaires post IPE course (47.1% overall response rate): 94 from the Faculty of Medicine, 37 from the School of Nursing, and 32 from the Faculty of Public Health. Using the unique identifiers present in the questionnaires we could only pair 111 pre and post questionnaires to study the impact of the course using paired analysis (Table 1).

**Table 1:** Distribution of questionnaires filled according to timing relative to the course and field of students- Number (column percent, reflecting response rate)

	Medicine (*N* = 180)	Nursing (*N* = 80)	Public Health (*N* = 86)	Total number
*Pre course*	139 (77%)	60 (75%)	61 (71%)	260
*Post course*	94 (52%)	37 (46%)	32 (37%)	163
*Paired questionnaires*	59 (33%)	28 (35%)	24 (28%)	111

Table 2 shows the mean change in students’ attitudes pre and post the IPE course, based on the paired analysis results of 111 pairs. The baseline mean scores of the 111 pairs were high across the five domains, with the patient centeredness domain having the highest mean score, and interprofessional biases having the lowest mean score. The paired analysis of the students pre and post IPE course showed an improvement in students’ attitudes towards the interprofessional biases domain only, however this improvement was not statistically significant. The results for the other four domains showed a slight decline in students’ attitudes. This decline was only statistically significant for the patient centeredness domain (Table 2).

**Table 2:** Paired analysis results of the mean change in scores of students’ attitudes pre and post IPE course (111 pairs)

	Pre course scores (Mean score)	Post course scores (Mean score)	Mean change (post - pre)	95% CI	*p*-value
Team roles and responsibilities	5.846	5.693	-0.153	[-0.323; 0.017]	0.078
Community Centeredness	6.261	6.198	-0.063	[-0.114;0.241]	0.481
Diversity and Ethics	6.567	6.414	-0.153	[-0.331;0.024]	0.091
Interprofessional Biases	4.117	4.270	0.153	[-0.057;0.364]	0.153
Patient Centeredness	6.594	6.315	-0.279	[-0.462;-0.096]	0.003**

Significance level *p* < 0.01**

## Discussion

The aim of the current study was to assess students’ attitudes towards interprofessional collaboration, before and after completing the IPEC course.

The baseline results with the highest mean for patient centeredness and lowest mean for interprofessional biases are comparable to those obtained from health sciences students (allied health, dentistry, medicine, nursing, pharmacy, public health, and social work) in Oklahoma university, where the lowest mean score was reported for interprofessional bias (4.8 ± 1.1 vs. 4.12 in our study) [14]. Similar to our study, the highest reported baseline mean score was for patient centeredness (6.6 ± 0.7) [14].

When comparing the pre and post IPE course survey results, the scores remained almost the same, with a slight decline post IPE course in four out of the five domains. A minor improvement was seen in the interprofessional biases domain. The survey scores were already elevated before the students took the IPE course. A study conducted on third year students from various health professions (allied health professionals, medicine, pharmacy, and nursing) in Dublin, Ireland showed similarly that the participants’ readiness and attitudes towards IPE were good pre workshop, and these continued to improve after the workshop [15]. The significant decline in students’ attitudes towards the patient centeredness domain reported in our population is similar to that found in another study conducted among pharmacy students using the IPAS tool, pre and post a three hour interprofessional forum [16]. Their results showed an increase in students’ scores on all scales except for the patient-centeredness domain [16].

## Limitations

Only 111 students completed the questionnaires before and after the course, therefore paired analyses were limited to less than 50% of students who took the IPEC course. Four domains of IPE (except for interprofessional biases) declined after the course, although the results failed to reach statistical significance, except for the patient-centered care domain. This could be attributed to the sample size or to the fact that the cases were theoretical. The students did not interact with patients in a real-life setting but rather discussed hypothetical cases.

## Strengths

We utilized the IPAS which is a validated instrument, with good factor structure and internal consistency [13]. The novelty of this tool is that it links the assessment of IPE to the IPEC report core competencies [13]. The diversity and ethics, community centeredness, and interprofessional biases are three domains unique to the IPAS [13]. In addition, our institution is one of very few that offer IPE to large groups of healthcare professionals in the country and the only one to our knowledge that has made IPE a requirement of the curriculum. A strength of this approach to teaching IPE through case-based discussion is the inclusion of both patient care (nursing and medicine) as well as population health (public health) students. IPE is often only taught to students of professions involving patient care. The question of whether the change of attitude toward interprofessional collaboration resulted in effective enhancement of collaboration after graduation needs to be evaluated.

## Conclusions

This study investigated baseline score and the impact of one IPE course on the attitude of students from different professions towards IPE. The results have shown elevated baseline scores, an improvement, although non-significant, in students’ attitudes towards the interprofessional biases domain, and no improvement in other domains after taking the course as compared to the baseline scores. These results suggests that further studies are needed to understand how IPE impacts students’ attitudes from different professions. As this study was carried out in one setting, we recommend that future studies are carried out on a larger sample size across different institutions, as well as to study the impact of such courses on the practices of health professionals.

## Data Availability

Data not available due to ethical restrictions but is available from the corresponding author on reasonable request.

## Abbreviations

IPE: Interprofessional education
AUB: American University of Beirut
IPEC: Interprofessional education and collaboration
IPAS: Interprofessional attitude scale
